# A model integrating donor gene polymorphisms predicts fibrosis after liver transplantation

**DOI:** 10.18632/aging.202302

**Published:** 2020-12-03

**Authors:** Chao Wang, Xueyou Zhang, Qi Ling, Shusen Zheng, Xiao Xu

**Affiliations:** 1 Department of Hepatobiliary and Pancreatic Surgery, Affiliated Hangzhou First People's Hospital, Zhejiang University School of Medicine, Hangzhou 310003, China; 2 Department of Hepatobiliary and Pancreatic Surgery, The First Affiliated Hospital, Zhejiang University School of Medicine, Hangzhou 310003, China; 3 NHC Key Laboratory of Combined Multi-Organ Transplantation, Hangzhou 310003, China

**Keywords:** liver transplantation, liver fibrosis, single nucleotide polymorphisms, novel integrated model

## Abstract

Post-transplant liver fibrosis (PTLF) is a common and severe complication in liver recipients. In this study, we assessed the impact of donor liver genetics on the development of PTLF. A total of 232 patients undergoing liver transplantation were included. Twenty-two single nucleotide polymorphisms (SNPs) associated with liver fibrosis were analyzed. Univariate analysis revealed seven donor SNPs to be associated with PTLF. In a multivariate analysis, independent risk factors of PTLF were genetic variation of donor GRP78 rs430397 (OR = 8.99, p = 0.003), GSTP1 rs1695 (OR = 0.13, p = 0.021), miRNA-196a rs12304647 (OR = 16.01, p =0.001), and TNF-α rs1800630 (OR = 79.78, p = 0.001); blood tacrolimus levels at maintenance > 7 ng/ml (OR =7.48, p <0.001); and post-transplant diabetes mellitus (OR = 7.50, p = 0.001). A predictive model that included donor SNPs showed better prognostic ability for PTLF than a model with only clinical parameters (AUROC: 0.863 vs 0.707, P < 0.001). Given that donor gene SNPs are associated with an increased risk of PTLF, this model integrated with donor gene polymorphisms may help clinicians predict PTLF.

## INTRODUCTION

Liver fibrosis (LF) can result from hepatic virus B (HBV) infection, excess alcohol-based liver disease, non-alcoholic fatty liver disease, autoimmune liver diseases, and hereditary diseases [1]. LF can progress to liver cirrhosis, which carries a risk for developing hepatocellular carcinoma (HCC) and other end-stage liver diseases.

Liver transplantation (LT) is a therapy for end-stage liver disease. However, HBV recurrence, diabetes, immunosuppressors, and alcohol intake after LT will cause LF again. Post-transplant liver fibrosis (PTLF) can also lead to new liver cirrhosis, which causes further life-threatening complications such as carcinoma or liver failure. Liver grafts also have poor survival due to the progression of liver fibrosis to graft cirrhosis [2]. Clinical research has shown that active HBV will drive liver inflammation and aggressive fibrogenesis [3]. Immunosuppressors such as tacrolimus advance fibrosis in liver transplant recipients in clinical trials [4]. Recent studies indicate that an intricate crosstalk between adipose tissue and the liver through adipokines and inflammatory cytokines is the mechanism for diabetes-induced liver fibrosis [5]. Alcohol intake will induce hepatocellular injury and activate hepatic stellate cells (HSCs), which leads to liver fibrogenesis [6].

Donor graft genetics play a vital role in LF and can reduce the survival of the graft as well as patient prognosis. Recent studies have shown that single nucleotide polymorphisms (SNPs) involved in liver fibrosis or cirrhosis is associated with the development of LF and may also increase PTLF occurrence. Those studies have identified a number of novel LF-susceptibility genes, including some inflammatory factors. Cytokines play an important role in mediating several immune responses, which are associated with an increased risk of LF. IL-1β is a proinflammatory cytokine and interleukin 1β (IL-1β) rs1143627 has functional importance in IL-1β biological activity [7]. Interleukin 10 (IL-10) rs1800872 mutations in the transcription start site (IL-10 promoter region) results in a lower concentration of IL-10 and a more vigorous immune response, which increases the risk for LF [8]. Tumor necrosis factor α (TNF-α) rs1800630 and rs1799724 mutations in gene promoter regions influence TNF-α expression at the transcriptional and post-transcriptional levels as well as increase susceptibility to LF [9–12]. Tumor necrosis factor β (TNF-β) rs909253 polymorphisms also promote pro-inflammatory activity related to hepatic inflammation that leads to LF [13]. Interleukin 4 (IL-4) rs2242350 CT and CC genotype frequencies were significantly higher in chronic hepatitis B patients with abnormal alanine aminotransferase (ALT) levels, associating them with liver inflammatory injury and LF [14].

Other research studies have also suggested that polymorphisms are responsible for LF. For example, the Cytochrome P4502E1 (CYP2E1) gene plays a key role in dimethylformamide metabolizing pathways. In CYP2E1 rs2031920, the minor T allele has a higher transcriptional and enzyme activity. Thus, this polymorphism may be a risk factor for LF [15]. The vitamin D receptor (VDR) is a nuclear hormone receptor that can act as a ligand-induced transcription factor. Gene variations of VDR rs7975232 at the 3′ end increases the susceptibility to liver fibrosis [16].

The GRP78 pathway is one of the most important responders to disease-associated stress. The glucose-regulated protein 78 (GRP78) rs430397 mutations lead to a variety of reactive oxygen species and cause LF [17, 18]. The human leukocyte antigen (HLA)-DQ rs2856718 predisposes the liver to chronic inflammation, which facilitates LF [19, 20]. Suriapranata et al. identified two SNPs in alpha-fetoprotein (AFP) intron 7 and 3′UTR—rs2298839 and rs10020432—as associated with increased fibrosis risk [21]. AFP rs10020432 at the 3’UTR of AFP is linked to miRNA gene expression regulation. The aberrant expression of miRNA promotes LF [22]. AFP rs2298839 is located in the gene intron 7 and creates a potential splice site that results in LF. The increase in the frequency of variant glutathione S-transferase p1 (GSTP1) rs1695 genotypes in LF patients when compared to non-LF controls is associated with LF development. The Ala to Val transition at codon 114 leads to significant differences in catalytic activity [23–25]. The matrix metalloproteinase 7(MMP-7) rs17884789 variant increases its distribution to the plasma membrane of HSCs and enhances cell migration. A recent study demonstrated that the MMP-7 variant can be a risk factor in the development of liver cirrhosis [26].

DNA repair mechanisms also play a role in LF development. Genetic variants of the human oxoguanine glycosylase 1 (hOGG1) rs1052133—important enzymes in the BER pathway—reduces DNA repair activity and increases the susceptibility to LF [27]. The X-ray repair cross-complementing gene 1 (XRCC1) is one of the molecules involved in DNA repair. An A to G transition of XRCC1 rs25487 results in a change from an arginine to glycine amino acid. There is evidence to suggest that this polymorphism influences the development of LF [28].

Li et al. verified that the programmed cell death-1 (PD1) rs10204525 polymorphisms were associated with altered circulating TNF-α and interferon (IFN) -γ levels in the liver, and caused LF in the Han Chinese [29]. ESR1 rs2077647, which is contained within a linkage disequilibrium block located in the estrogen receptor α (ESR1) promoter region to intron 3, influenced susceptibility to LF [30]. Xiao et al. revealed that polymorphism in the core promoter region of Angiotensinogen 6 (AGT-6) rs5051 determines LF progression [31]. Polymorphisms in microRNA may change its function. Evidence has revealed the association between miRNA-196a rs12304647 and the occurrence of LF in HBV patients [32, 33]. Notably, Peng et al. identified the antizyme inhibitor 1 (AZIN1) rs2679757 and the transient receptor potential cation channel subfamily member 5 (TRPM5) rs886277 [34] were associated with the risk for HBV-related liver cirrhosis in Chinese people. The genes may also be involved in liver fibrosis pathogenesis.

However, the impact of liver allograft genetic polymorphisms on graft fibrosis has not been fully investigated. The aim of this study was to investigate whether the 23 donor SNPs associated with liver fibrosis affects PTLF development.

## RESULTS

### PTLF clinical characteristics

The incidence rate of PTLF was 18.1% (42/232). Clinical characteristic comparisons between the LF and the non-LF groups are shown in Table 1. Significant differences between the two groups included: Recipient age (P = 0.004), body mass index (BMI, P = 0.003), recipient primary disease (HCC or not, P = 0.028), tacrolimus level at maintenance (P = 0.005), and post-transplant diabetes mellitus (PTDM, P = 0.01).

**Table 1:** Donor and recipient characteristics.

**Donor characteristic**	**fibrosis (n = 42)**	**Non-Fibrosis (n = 190)**	***P***
Age (yr)	35.1 ± 7.0	34.3 ± 7.5	0.501
Male	40	144	0.678
BMI (kg/m^2^)	21.7 ± 1.4	22.3 ± 1.6	0.106
ABO Incompatible	4	28	0.253
Cause of death			
Trauma	27	100	0.17
CVA	15	90
HBsAg positive	3	20	0.507
Anti-HBcAb positive	8	30	0.606
CIT (hr)	7.9 ± 4.3	7.9 ± 3.8	0.998
DWIT (min) ^a^	7.4 ± 8.3	6.6 ±6.7	0.526
UW (vs. others ^b^)	40	178	0.702
**Recipient characteristic**			
Age (yr)	49.8 ± 9.3	44.7 ± 10.3	**0.004**
Male	32	162	0.151
BMI (kg/m^2^)	21.8 ± 2.9	22.5 ± 3.5	**0.003**
MELD score	18.6 ± 7.9	18.7 ± 9.4	0.952
Child score	9.5 ± 2.0	9.1 ± 2.4	0.288
HCC	80	10	**0.028**
Cirrhosis	34	166	0.275
Acute liver failure	4	6	0.066
HBsAg positive	38	166	0.576
Anti-HBcAb positive	30	130	0.054
Immunosuppressant			
Corticosteroid single-pulse	22	122	0.153
SIR	2	22	0.189
TAC	40	168	0.189
TAC at 6-month (ng/ml)	7.3 ± 2.5	7.2 ± 2.5	0.902
TAC at maintenance (ng/ml)	6.8 ± 2.6	5.4 ± 2.2	**0.005**
Post-transplant complications			
HBV recurrence	2	24	0.143
Hyperlipidemia	2	8	0.873
PTDM	10	18	**0.010**
Vascular complication	6	20	0.485

^a^: includes only DCD donors. ^b^: other preservation solution includes histidine-tryptophan-ketoglutarate (HTK) and Celsior; BMI: body mass index; CVA: cerebrovascular accident; CIT: Cold ischemia time; DWIT: donor warm ischemia time; UW: university of Wisconsin; MELD: model for end-stage liver disease; HCC: hepatocellular carcinoma; TAC; tacrolimus; SIR: sirolimus; HBV: hepatitis B virus; PTDM: post-transplant diabetes mellitus.

### Association between donor gene polymorphism and PTLF

The distribution of donor SNPs associated with PTLF is shown in Table 2. Only those SNPs’ in Hardy-Weinberg equilibrium (P > 0.05, Supplementary Table 1) were analyzed. Among the 23 SNPs, seven were different between the LF and non-LF groups. Minor allele frequency (MAF) of donor SNPs TNF-β rs909253, HLA-DQ rs2856718, hOGG1 rs1052133, and GSTP1 rs1695 were 0.5, 0.34, 0.39, and 0.16, respectively. MAF of donor GRP78 rs430397, miRNA-196a rs12304647, and TNF-α rs1800630 was 0.16, 0.26, and 0.17, respectively.

**Table 2:** Distribution of donor SNPs associated with fibrosis after liver transplantation.

**Donor SNP**	**Genotype**	**Fibrosis**	**Non-fibrosis**	**OR**	**95% CI**	P^*^
Rs430397	C/C	138 (72.6%)	24 (57.1%)	1.00		
	C/T	46 (24.2%)	18 (42.9%)	2.25	(1.12-4.51)	0.020
	T/T	6 (3.2%)	0 (0%)	0.85	0.80-0.91	0.309
	C/C	138 (72.6%)	24 (57.1%)	1.00		
	C/T-T/T	52 (27.4%)	18 (42.9%)	1.99	(1.00-3.97)	0.053
Rs909253	G/G	48 (25.3%)	8 (19.1%)	1.00		
	G/A	108 (56.8%)	14 (33.3%)	0.78	(0.31-1.98)	0.597
	A/A	34 (17.9%)	20 (47.6%)	3.53	(1.39-8.95)	0.006
	G/G-G/A	156 (82.1%)	22 (52.4%)	1.00		<0.001
	A/A	34 (17.9%)	20 (47.6%)	4.17	(2.05-8.49)
Rs2856718	T/T	84 (44.2%)	26 (61.9%)	1.00		
	C/T	74 (39%)	12 (28.6%)	0.52	(0.25-1.11)	0.089
	C/C	32 (16.8%)	4 (9.5%)	0.40	(0.13-1.25)	0.106
	T/T	84 (44.2%)	26 (61.9%)	1.00		
	C/T-C/C	106 (55.8%)	16 (38.1%)	0.49	(0.25-0.97)	0.037
Rs1052133	G/G	82 (43.2%)	6 (14.3%)	1.00		
	C/G	78 (41%)	28 (66.7%)	4.91	(1.93-12.49)	<0.001
	C/C	30 (15.8%)	8 (19.1%)	3.64	(1.17-11.37)	0.020
	G/G	82 (43.2%)	6 (14.3%)	1.00		
	C/G-C/C	108 (56.8%)	36 (85.7%)	4.56	(1.83-11.33)	0.001
Rs1695	A/A	126 (66.3%)	34 (81%)	1.00		
	G/A	62 (32.6%)	6 (14.3%)	0.36	(0.14-0.90)	0.020
	G/G	2 (1.1%)	2 (4.8%)	3.71	(0.50-27.28)	0.170
	A/A	126 (66.3%)	34 (81%)	1.00		
	G/A-G/G	64 (33.7%)	8 (19.1%)	0.46	(0.20-1.06)	0.054
Rs12304647	A/A	110 (57.9%)	16 (38.1%)	1.00		
	C/A	68 (35.8%)	22 (52.4%)	2.22	(1.09-4.53)	0.025
	C/C	12 (6.3%)	4 (9.5%)	2.29	(0.66-7.98)	0.183
	A/A	110 (57.9%)	16 (38.1%)	1.00		0.020
	C/A-C/C	80 (42.1%)	26 (61.9%)	2.23	(1.13-4.44)	
Rs1800630	C/C	140 (73.7%)	24 (57.1%)	1.00		
	C/A	44 (23.2%)	14 (33.3%)	1.86	(0.88-3.89)	0.099
	A/A	6 (3.2%)	4 (9.5%)	3.89	(1.02-14.81)	0.034
	C/C	140 (73.7%)	24 (57.1%)	1.00		
	C/A-A/A	50 (26.3%)	18 (42.9%)	2.10	(1.05-4.19)	0.033

SNP: single-nucleotide polymorphism; OR: odds ratio; CI: confidence interval. p* value was calculated from Chi-square test.

The donor liver graft with GRP78 rs430397, TNF-β rs909253, hOGG1 rs1052133, miRNA-196a rs12304647, and TNF-α rs1800630 polymorphism showed a remarkably higher risk of PTLF. LT patients with donor liver graft GSTP1 rs1695 and HLA-DQ rs2856718 polymorphisms had a reduced risk of PTLF (Table 2).

### Risk factors for PTLF: A multivariate logistic regression model

Clinical values and donor SNPs that showed statistical significance in univariate analysis (Table 2) were analyzed in multivariate analysis. Tacrolimus levels > 7 ng/ml at maintenance after LT, PTDM, donor GRP78 rs430397, GSTP1 rs1695, miRNA-196a rs12304647, and TNF-α rs1800630 genotypes were independent risk factors of PTLF (Table 3).

**Table 3:** The risk factors of fibrosis after liver transplantation.

**Donor SNP characteristic**		**Univariate**		**Multivariate**	
		**OR (95% CI)**	P*	**OR (95% CI)**	***P^#^***
rs430397	C/C	/	/	8.99 (2.16-37.56)	0.003
	C/T	2.89 (1.45-5.79)	0.003		
	T/T	/	1		
rs909253	G/G	/	/	/	/
	G/A	0.30 (0.14-0.65)	0.002	/	/
	A/A	0.33 (0.13-0.85)	0.022	/	/
rs2856718	T/T	/	/	/	/
	C/T	1.30 (0.39-4.33)	0.672	/	/
	C/C	2.48 (0.80-7.66)	0.115	/	/
rs1052133	G/G	/	/	/	/
	C/G	1.01 (0.43-2.33)	0.99	2.69 (0.59-13.16)	0.223
	C/C	0.13 (0.04-0.46)	0.001	0.19 (0.03-1.35)	0.098
rs1695	A/A	/	/		
	G/A	0.36 (0.14-0.90)	0.029	0.13 (0.02-0.74)	0.021
	G/G	3.71 (0.50-27.28)	0.198		1
rs12304647	A/A	/	/		
	A/C	2.91 (1.41-6.01)	0.004	16.01 (3.16-81.16)	0.001
	C/C	2.67 (0.76-9.41)	0.127	5.05 (0.62-40.85)	0.129
rs1800630	C/C	/	/		
	C/A	1.86 (0.89-3.89)	0.102	1.60 (0.42-6.07)	0.486
	A/A	3.89 (1.02-14.81)	0.047	79.78 (6.38-998.12)	0.001
**Recipient characteristic**					
Age >55 yr		3.06 (1.37-6.82)	0.006		
TAC at maintenance >7 ng/ml		3.98 (1.70-9.33)	0.002	23.36 (4.71-115.75)	<0.001
HCC		0.43 (0.20-0.93)	0.031		
PTDM		2.99 (1.26-7.06)	0.013	5.36 (1.05-27.51)	0.044
BMI >24		0.44 (0.19-1.01)	0.053		

CI: confidence interval; TAC: tacrolimus; HCC: hepatocellular carcinoma; PTDM: post-transplant diabetes mellitus; SNP: single nucleotide polymorphism; BMI: body mass index. p* value was calculated from univariate logistic regression test; p^#^ value was calculated from multivariate logistic regression test.

### The value of risk factors and models to predict PTLF

**Figure 1:**
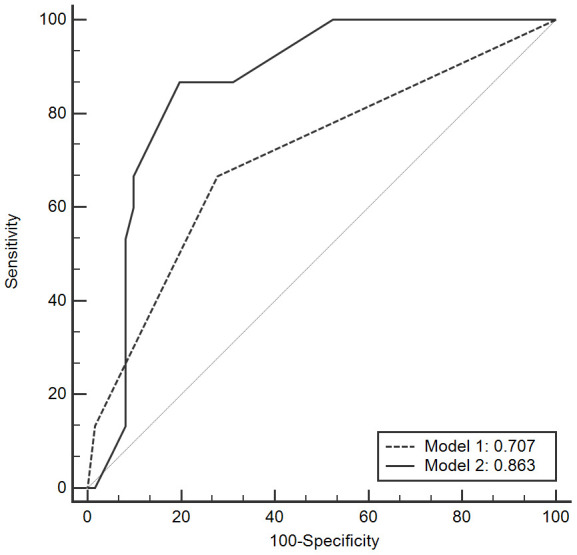
**ROC curves of the model to predict LF after LT.** LF: liver fibrosis; LT: liver transplantation; ROC: receiver operating characteristic curve.

The predictive capacity of the risk factors on PTLF was further assessed by logistic regression analysis. Different predictive models were established by different inclusion criteria. Model 1 only included clinical values while Model 2 contained donor genetic mutations as risk factors. AUROC was used to evaluate the model’s predictive power on PTLF (Figure 1).

Model 2 demonstrated better predictive power than Model 1 by using AUROC (0.863 vs. 0.707, P < 0.001, Table 4). This means that models with donor SNPs variants could improve the ability to predict PTLF when compared to models that only contain clinical parameters.

**Table 4:** AUROC of the risk model associated with liver fibrosis after liver transplantation.

	**AUROC predictive value**	**95% CI**
Model 1		
Recipient TAC at maintenance >7 ng/ml	0.707	0.628-0.778
PTDM		
Model 2		
Recipient TAC at maintenance >7 ng/ml		
PTDM		
Donor rs430397 CT genotype	0.863	0.798-0.913
Donor rs1695 GA genotype		
Donor rs12304647 AC genotype		
Donor rs1800630 AA genotype		

CI: confidence interval; AUROC: area under the receiver operating characteristic curve; PTDM: post-transplant diabetes mellitus; TAC: tacrolimus.

## DISCUSSION

Our results show that donor genetic backgrounds influence PTLF development in liver recipients. Seven SNPs played significant roles in the occurrence of PTLF: GRP78 rs430397, TNF-β rs909253, TNF-α rs1800630, HLA-DQ rs2856718, hOGG1 rs1052133, GSTP1 rs1695, and miRNA-196a rs12304647. Rs430397 is located in intron 5 of the GRP78 gene and may be related to mitogenic response and stress [17]. GRP78 can adjust the activation of ER stress transducers such as IRE1, PERK, and ATF6 [18]. In this study, LT patients with liver fibrosis were more likely to have rs430397 CT genotypes in univariate and multivariate logistic analysis. TNF-α is a proinflammatory cytokine and uninfected “bystander” cells. TNF-α and TNF-β can activate the transcription factor NF-kB, promoting pro-inflammatory genes that are related to hepatic inflammation, which leads to LF [12, 13]. TNF-α gene polymorphisms may also be involved in NAFLD progression [9], which is associated with PTLF occurrence. In this study, we found that TNF-β rs909253 GA and AA genotypes increased LF susceptibility by univariate analysis. Multiple logistic regression analysis showed that the rs909253 genotype had no association with PTLF. Unlike rs909253, the TNF-α rs1800630 AA genotype increased LF susceptibility after LT by both univariate and multiple analysis. Taken together, these findings suggest that rs1800630 may increase PTLF occurrence.

The rs2856718 is located in the intergenic region between HLA-DQA2 and HLA-DQB. Recent Genome-Wide Association Studies (GWAS) containing Japanese samples showed that rs2856718 within the HLA-DQ locus was related to hepatitis B persistence [19]. The C genotype is also involved in HBV elimination when compared to the A genotype [20]. This indicates that a single rs2856718 C may decrease the risk of HBV infection and LF. Similarly, using the Chi-square test we found that patients with a C allele have a reduced risk of PTLF. The hOGG1 protein catalyzes the resection of 8-oxoG from DNA. Rs1052133 polymorphisms leads to substitution from serine to cysteine at codon 326, and can reduce DNA repair activity [27]. We found that patients with CC genotypes were linked to lower risk (0.13 fold) of PTLF than those with GG genotypes. Unfortunately, this result was not observed in the multiple logistic analysis.

GSTP1, a member of the GST superfamily, impacts hepatic conditions in patients with cryptogenic cirrhosis because of its expression in the biliary epithelium. Rs1695 has been found in GSTP1 genes, some of which increased the occurrence of liver cirrhosis [23]. This study suggests from univariate and multivariate analysis that individuals who carry the GA genotype may have a reduced risk of PTLF occurrence. Our results are in contrast to a previous study which indicated an increased risk of LF for GSTP1 genotype mutations [23–25]. This discrepancy may be caused by the gene-environment interaction, such as using immunosuppressors, after LT. Polymorphisms in microRNA may change the expression and function of microRNAs and some studies have demonstrated that rs12304647 polymorphisms increase liver cirrhosis occurrence [32, 33]. This study showed that patients with an AC genotype had an increased of PTLF. Furthermore, a significant association between AC genotype rs12304647 and LF were seen under the multiple regression model.

In addition, there was crosstalk between the transplanted liver and recipient. PTDM—especially type 2 diabetes mellitus (T2DM)—has been demonstrated to increase LF occurrence [35]. T2DM patients with an unusually high aortic stiffness are also at an abnormal risk for developing liver disease. DM contributes to advanced glycation end-products, which can affect the structure and function of extracellular matrix proteins (collagen and elastin), changing their physical properties and leading to additional fibrosis [35]. We observed that patients with PTDM increased PTLF risk in both univariate and multiple analysis. Tacrolimus is a proven safe and effective immunosuppressive drug for LT [36], but has the possibility of progressing LF, especially in LT patients who received steroids [37]. We also found that tacrolimus might increase LF risk in patients who received LT.

The clinical status, such as blood tacrolimus concentration and PTDM, can injure the liver and lead to LF. We found that genetic mutations of the donor liver rs430397, rs1695, rs12304647, and rs1800630 could also result in LF occurrence. Several studies have shown that gene polymorphisms are involved in increasing the rate of LF occurrence in humans [10, 27, 32, 33]. A model with two clinical parameters (Model 1) showed a moderate predictive power of LF. Model 2 included donor gene polymorphisms and showed significantly greater predictive power than Model 1. These results indicate that the donor graft could take on LF-susceptibility polymorphisms, which could increase PTLF occurrence.

One limitation of the study is that it was based on a relatively small number of Han Chinese samples, and the majority of individuals had HBV-related liver disease. This research should be validated in a large-sample size study with multiple etiologies of liver diseases, such as hepatitis C virus, NAFLD, and alcohol. Another limitation of this analysis was the diagnosis of LF using liver biopsy, which is the standard method for assessing fibrosis, but is not favored by recipients due its invasive nature. Nonetheless, we are optimistic that new onsets of early warning predictors of fibrosis can be found in the future using non-invasive techniques, allowing precise assessment of fibrosis in individuals after LT.

## CONCLUSIONS

We demonstrated that donor rs430397, rs1695, rs12304647, and rs1800630 polymorphisms are associated with an increase of PTLF and can serve as potential clinical biomarkers for the prediction of LF. For patients with donor livers, those genetic variants, blood tacrolimus concentrations at maintenance, and PTDM may also contribute to PTLF. Furthermore, the integrated model with donor gene polymorphisms can be a potential tool for predicting PTLF.

## MATERIALS AND METHODS

### Study subjects

We prospectively enrolled 328 adult patients who underwent LT due to HBV-related diseases. The donor deaths occurred in our center between October 2015 and October 2017. Liver recipients who passed away or had graft failure within two years after LT were excluded. Those with biliary complication-induced fibrosis at the two year check point were also excluded. This resulted in 232 patients that were included in our study and their characteristics are shown in Table 1. All patients received the standard protocol (nucleoside analogue plus low-dose hepatitis B immunoglobulin) to prevent HBV recurrence and were followed up routinely [38].

This study was approved by the Ethics Committee of The First Affiliated Hospital of Zhejiang University on the basis of the Regulations on Human Organ Transplant and national legal requirements. This study complies with the guidelines of China’s Ethical Committee and the Helsinki Declaration. All donors and recipients gave informed consent before transplantation. No donor organs were obtained from executed prisoners.

The end point of this study was taken two years after LT and assessed graft fibrosis by using the fibrosis-4 index (FIB-4) as well as the aspartate aminotransferase -to-platelet ratio index (APRI) score [39, 40].

### Data collection

Data were collected and analyzed for patient age, gender, body mass index (BMI), blood type, ischemia time, recipient primary disease (HCC or not), comorbidities, immunosuppressive protocol, post-transplant complications, and mortality.

### Definition

The criterion for PTLF is both an APRI score >1.50 and FIB-4 score >3.25. The FIB-4 values were defined as the formula age (years) × AST [U/l]/(platelets [10^9^/L] × (ALT [U/L])1/2) [39]. The APRI values were calculated by the formula AST/upper limit of normal]/platelet count [10^9^/L] × 100 [40].

### Genotyping

Genomic DNA was extracted from freshly frozen donor liver tissues and SNPs were detected using Applied Biosystems SnaPShot technology as mentioned previously [41]. Twenty-two fibrosis-associated SNPs in fifteen genes were chosen (GRP78 rs430397, IL-10 rs1800872, IL-1β rs1143627, ESR-1 rs2077647, MMP7 rs17884789, IL-4 rs2243250, VDR rs7975232, TNF-β rs909253, AGT-6 rs5051, PD-1 rs10204525, HLA-DQ rs2856718, CYP2E1 rs2031920, hOGG1 rs1052133, XRCC1 rs25487, AFP rs2298839, AFP rs10020432, GSTP1 rs1695, miRNA-196a rs12304647, TNF-α rs1799724, TNF-α rs1800630, AZIN1 rs2679757, and TRPM5 rs886277, Supplementary Table 1).

### Statistical analysis

Quantitative variables are presented as the mean ± SD or median (interquartile range, IQR) and were compared using the Student’s t-test or Mann-Whitney test. Categorical variables were presented as values (percentages) and compared using the Chi-square test. Cumulative survival comparison was performed using the Kaplan-Meier method. Risk factors were identified using logistic regression analysis. Variables selected for univariate analysis were entered into a step-by-step multivariate regression model. A P value of < 0.05 was regarded as statistically significant. Data analysis was performed using SPSS version 23.0 (SPSS Inc, Chicago, IL) and SNP States (https://www.snpstats.net/snpstats/start.htm?q=snpstats/start.htm). A P value < 0.05 was considered statistically significant. The predictive model instruction and risk score calculation are described in our previous research [41]. The area under the receiver operating characteristic curve (AUROC) was calculated to evaluate the predictive ability of the PTLF model. An AUROC of 0.5 shows a lack of discriminatory ability, and 1 indicates perfect discrimination.

## Supplementary Material

Supplementary Table 1
