# Supplementary material for: A model integrating donor gene polymorphisms predicts fibrosis after liver transplantation
Source: Aging (Albany NY). 2020 Dec 3;13(1):1264–75. doi: 10.18632/aging.202302 (PMC7835018; doi:10.18632/aging.202302)
Supplement: Supplementary Table 1 [file aging-13-202302-s001.pdf]

SUPPLEMENTARY TABLE

Supplementary Table 1. Allele frequency and Hardy-Weinberg equilibrium of SNPs.

SNP	1000Genomes		This study		HWE *P**
	Major/minor Allele	MAF	Minor Allele	MAF	
**rs430397**	C/T	0.134	T	0.16	0.91
rs1800872	C/T	0.435	C	0.28	0.90
rs1143627	G/A	0.472	A	0.5	0.18
rs2077647	T/C	0.467	C	0.42	0.69
rs17884789	C/T	0.022	T	0.1	0.09
rs2243250	C/T	0.469	C	0.25	0.86
rs7975232	A/C	0.485	A	0.33	0.90
**rs909253**	A/G	0.389	G	0.5	0.43
rs5051	T/C	0.289	C	0.21	0.11
rs10204525	C/T	0.351	C	0.3	0.86
**rs2856718**	C/T	0.423	C	0.34	0.007
**rs1052133**	C/G	0.302	C	0.39	0.52
rs2298839	G/A	0.488	A	0.31	0.36
rs10020432	G/A	0.471	A	0.32	0.20
**rs1695**	A/G	0.353	G	0.16	0.28
**rs12304647**	A/C	0.299	C	0.26	0.98
rs25487	C/T	0.317	T	0.23	0.20
**rs1800630**	C/A	0.154	A	0.17	0.08
rs2679757	A/G	0.333	G	0.26	0.60
rs886277	C/T	0.464	T	0.35	0.14
rs2031920	C/T	0.07	T	0.17	0.11
rs1799724	C/T	0.10	T	0.09	0.001

MAF: Minor allele frequency; HWE: Hardy-Weinberg equilibrium; SNP: single-nucleotide polymorphism. *: P value was calculated from the Pearson χ^2 test.
